# High rate of seroeligibility among *MYBPC3*-associated hypertrophic cardiomyopathy patients for TN-201, an adeno-associated virus serotype 9 *MYBPC3* gene therapy

**DOI:** 10.3389/fmed.2025.1635586

**Published:** 2025-09-12

**Authors:** Milind Y. Desai, Daniele Massera, Heng Wang, Timothy C. Wong, Omar Wever-Pinzon, Neal K. Lakdawala, John R. Giudicessi, Theodore Abraham, Sherif Nagueh, Florian Rader, Ahmad Masri, Emre Turer, Pfumo Mushonga, Elizabeth Butala Flores, William Harrison, Natalia Sonicheva-Paterson, Gretchen Argast

**Affiliations:** ^1^Cleveland Clinic Foundation, Cleveland, OH, United States; ^2^NYU Langone, New York, NY, United States; ^3^DDC Clinic for Special Needs Children, Middlefield, OH, United States; ^4^UPMC Hypertrophic Cardiomyopathy Center, University of Pittsburgh, Pittsburgh, PA, United States; ^5^Advanced Heart Failure Treatment and Recovery Section, University of Utah Health Sciences Center, Salt Lake City, UT, United States; ^6^Brigham and Women’s Hospital, Boston, MA, United States; ^7^Mayo Clinic, Rochester, MN, United States; ^8^UCSF HCM Center of Excellence, University of California, San Francisco, San Francisco, CA, United States; ^9^Houston Methodist, Houston, TX, United States; ^10^Cedars-Sinai Medical Center, West Hollywood, CA, United States; ^11^Oregon Health and Science University School of Medicine, Portland, OR, United States; ^12^The University of Texas Southwestern Medical Center, Dallas, TX, United States; ^13^Bioagilatyx, NC, United States; ^14^Tenaya Therapeutics, South San Francisco, CA, United States

**Keywords:** MYBPC3, seroprevalence, HCM, adenoassociated virus 9, gene therapy

## Abstract

**Background:**

The genetic etiology of hypertrophic cardiomyopathy (HCM) and the critical role of sarcomeric variants in its pathogenesis are well recognized (1). Among these, loss-of-function variants in the myosin binding protein C gene (*MYBPC3*) are the most prevalent, resulting in protein insufficiency when compared to healthy controls (1). Preclinical studies have shown that recombinant adeno-associated virus serotype 9 (rAAV9) carrying the full-length *MYBPC3* transgene can increase protein expression and improve cardiac function. However, pre-existing anti-AAV9 antibodies—neutralizing (NAb) and total (TAb)—may limit gene transfer efficacy and eligibility for gene therapy. We sought to evaluate the prevalence of anti-AAV9 antibodies in patients with *MYBPC3*-associated HCM to optimize patient selection for MyPEAK-1, an ongoing trial evaluating the safety, tolerability, and pharmacodynamics of TN-201, an AAV9: *MYBPC3* gene therapy.

**Methods:**

This was a prospective, cross-sectional study of 100 adults (aged 18–65 years) with symptomatic *MYBPC3*-associated HCM (NYHA II–IV). Blood samples were analyzed for anti-AAV9 NAb (transduction inhibition assay) and TAb (electrochemiluminescence assay). Titers ≥1:10 were considered positive. Associations between antibody levels and demographic and clinical characteristics were explored using statistical tests.

**Results:**

Pre-existing anti-AAV9 NAb were undetectable in 50% of patients. Among those with detectable titers (range: 1:10–1:720), only 16% exceeded 1:40. TAb were undetectable in 53%; titers ranged from 1:10 to 1:65,600. A strong correlation was observed between NAb and TAb titers (r = 0.671, *p* < 0.001). Serostatus was not significantly associated with age, sex, NYHA class, or ethnicity (all *p* > 0.05).

**Conclusion:**

Pre-existing immunity to AAV9 was absent or low in most *MYBPC3*-associated HCM patients, with only a small proportion exceeding standard NAb thresholds for AAV gene therapy trials. These findings support the feasibility of a clinical trial of TN-201, an AAV9-based *MYBPC3* gene replacement therapy, in this population. Given the concordance between NAb and TAb assays, either may be suitable for screening.

## Introduction

The genetic etiology of hypertrophic cardiomyopathy (HCM) and the critical role of sarcomeric variants in its pathogenesis are well-recognized (1). Among these, loss-of-function variants in the myosin binding protein C gene (*MYBPC3*) are the most prevalent, resulting in ~60% of the normal protein levels necessary for optimal cardiac function (1). TN-201 is an adeno-associated virus serotype 9 (AAV9)-based gene therapy designed to deliver a working *MYBPC3* gene to heart muscle cells via a single intravenous infusion, increasing MyBP-C protein levels to address the underlying cause of *MYBPC3*-associated HCM with the aim of halting or even reversing disease after a single dose. Preclinical *in vivo* and *in vitro* studies have demonstrated that administration of a recombinant adeno-associated virus serotype 9 (rAAV9) carrying the full-length *MYBPC3* transgene restored missing protein and improved cardiac function, offering a strong rationale for a gene therapy to reverse disease progression, potentially in *MYBPC3*-associated HCM (2).

The first interim clinical data from the TN-201 gene therapy trial—designed to evaluate the safety, tolerability, pharmacodynamics, and efficacy of TN-201—show promising results. In the initial three patients who received a one-time infusion of TN-201 at the 3E13 vg/kg dose, the therapy demonstrated a compelling safety profile, robust cardiac transduction, encouraging levels of *MYBPC3* expression, and early signs of clinical improvement or disease stability, all of which represent positive early signals of clinical impact of TN-201 (3, 4).

Adeno-associated viruses (AAVs) are naturally occurring and non-pathogenic. AAV9 has shown relatively high affinity for cardiomyocytes among all serotypes. However, pre-existing immunity to AAVs, manifesting as neutralizing (NAb) or total (TAb) antibodies, may impact vector transduction efficiency or possibly increase the risk of an immune response following administration of an AAV-based gene therapy. Therefore, neutralizing antibodies may represent a major barrier in gene transfer, which could potentially result in clearance of the vector before it reaches the target cell (5, 6). The impact of preexisting immunity suggests that screening patients for serostatus may help identify those most likely to benefit from gene transfer. Rare, pre-existing antibodies may increase the risk of immune responses post-treatment, such as complement activation, hepatotoxicity, and inflammatory responses; however, serious AAV-related immune events are uncommon in clinical trials (7–11).

Prior studies in patients, including those with heart failure, showed increasing AAV seroprevalence with older age (12, 13). Since the *MYBPC3*-associated HCM population is, on average, older than all other populations enrolled in intravenously administered AAV9 gene therapy programs, it was expected that more patients may not be eligible for AAV gene therapy due to pre-existing immunity to the serotype than expected (14). Although AAV9 has not been evaluated as extensively as AAV2 for pre-existing NAb, several studies suggest a 30–60% seroprevalence reported by others across various populations (Table 1).

**Table 1:** Baseline demographics and medical history of study population.

Characteristic	Total (*N* = 100)
Age (years)	
Median (Min–Max)	48.0 (19–65)
Age Groups (years) [*n* (%)]	
≥18 to <30	19 (19.0)
≥30 to <40	14 (14.0)
≥40 to <50	23 (23.0)
≥50 to ≤65	44 (44.0)
Sex [*n* (%)]	
Female	34 (34.0)
Male	66 (66.0)
Race [*n* (%)]	
American Indian or Alaska Native	0
Asian	3 (3.0)
Black or African American	10 (10.0)
Native Hawaiian or Other Pacific Islander	1 (1.0)
White	80 (80.0)
Not reported/Unknown	6 (6.0)
Ethnicity [*n* (%)]	
Hispanic or Latino	5 (5.0)
Not Hispanic or Latino	92 (92.0)
Not reported/Unknown	3 (3.0)
*MYBPC3* variant [*n* (%)]	
Pathogenic/likely pathogenic (P/LP), truncating	100 (100)
Additional sarcomeric P/LP variant	11 (11.0)
HCM diagnosis [*n* (%)]	
Obstructive	44 (44.0)
Nonobstructive	48 (48.0)
Non-obstructive status post-SRT	8 (8.0)
Implantable cardiac defibrillator	56 (56.0)
New York Heart Association Class [*n* (%)]	
Class I	0
Class II	87 (87.0)
Class III	13 (13.0)
Septal myectomy	9 (9.0)

HCM, hypertrophic cardiomyopathy; MYBPC3, myosin-binding protein C gene; AAV9, adeno-associated virus serotype 9.

We sought to evaluate the prevalence of pre-existing immunity to AAV9 in *MYBPC3*-associated HCM to estimate the seroeligibility of *MYBPC3* + HCM patients and support patient selection for MyPEAK-1 (NCT05836259), a trial evaluating the safety, tolerability, and pharmacodynamics of TN-201, an AAV9 *MYBPC3* gene therapy (4).

## Methods

This was a prospective, cross-sectional, noninterventional, multicenter (*n* = 12) cohort study. The inclusion criteria were the following: symptomatic adult patients (New York Heart Association [NYHA] class II-IV), aged 18–65, with HCM diagnosis and confirmed pathogenic or likely pathogenic (P/LP) *MYBPC3* truncating variant. Patients were excluded if they were receiving immunomodulating therapy or chemotherapy, had a history of clinically significant liver disease, or had prior exposure to gene therapy. All patients attended a single study visit where a blood sample was collected to assess the presence of anti-AAV9 antibodies. No investigational product was administered. Institutional review board approval was obtained, and all patients signed informed consent.

### NAb assay

AAV9 NAb were assessed by a transduction inhibition assay (BioAgilytix, NC, USA). Chinese hamster ovary (CHO) Lec2 cells were incubated with a mixture of participant serum (minimum dilution 2) and a custom AAV9 virus with luminescent reporter gene (multiplicity of infection 6,000). Luminescence was measured after 24 h of incubation at 37 °C (One-Glo, Promega). Luminescence from each well was normalized to the plate negative control and compared to a statistically established cut-off point for positivity (1% false positive rate, FPR). Titer was determined through serial dilution of participant serum. Results were reported as not detected (titer <1:10) and detected (≥1:10), with titer when present.

### TAb assay

AAV9 TAb was detected by electrochemiluminescent (ECL) bridging assay (BioAgilytix, NC, USA). Assay plates were coated with AAV9 capsid (Tenaya Therapeutics, Inc.). Participant serum was diluted 10-fold and incubated on the plate for 1 h at room temperature to bind TAb. Labeled AAV9 capsid (Tenaya Therapeutics) was added to detect the bound TAb through ECL reaction (Meso Scale Diagnostics). Sample luminescence was normalized to the plate negative control and compared to a statistically determined cut-off point for positivity (1% FPR). Titer was determined through serial dilution of participant serum. Results were reported as not detected (<1:10) and detected (≥1:10), with titer when present.

### Statistical analysis

Patients’ demographic and baseline characteristics were summarized using descriptive statistics. Medical history included NYHA class, ICD placement, and type of HCM (obstructive, non-obstructive, or non-obstructive post-myectomy). NAb and TAb titer values were provided for samples with detectable antibody levels. To assess the relationship between NAb and TAb titers, Pearson’s correlation coefficient was calculated. Subgroup analyses were conducted to explore potential associations between pre-existing immunity to AAV9 (as measured by TAb and NAb titers) and key demographic factors. Comparison between subgroups was performed using the Chi-square test or Fisher’s exact test, as appropriate. A *p*-value of <0.05 was considered significant.

## Results

A total of 100 adult HCM patients (aged 18–65 years) were enrolled in the study (Table 1). All participants were symptomatic (NYHA class II or III) and had *MYBPC3* P/LP truncating variants; 44% had obstructive HCM, and ICD placement in 55% of patients.

**Figure 1:**
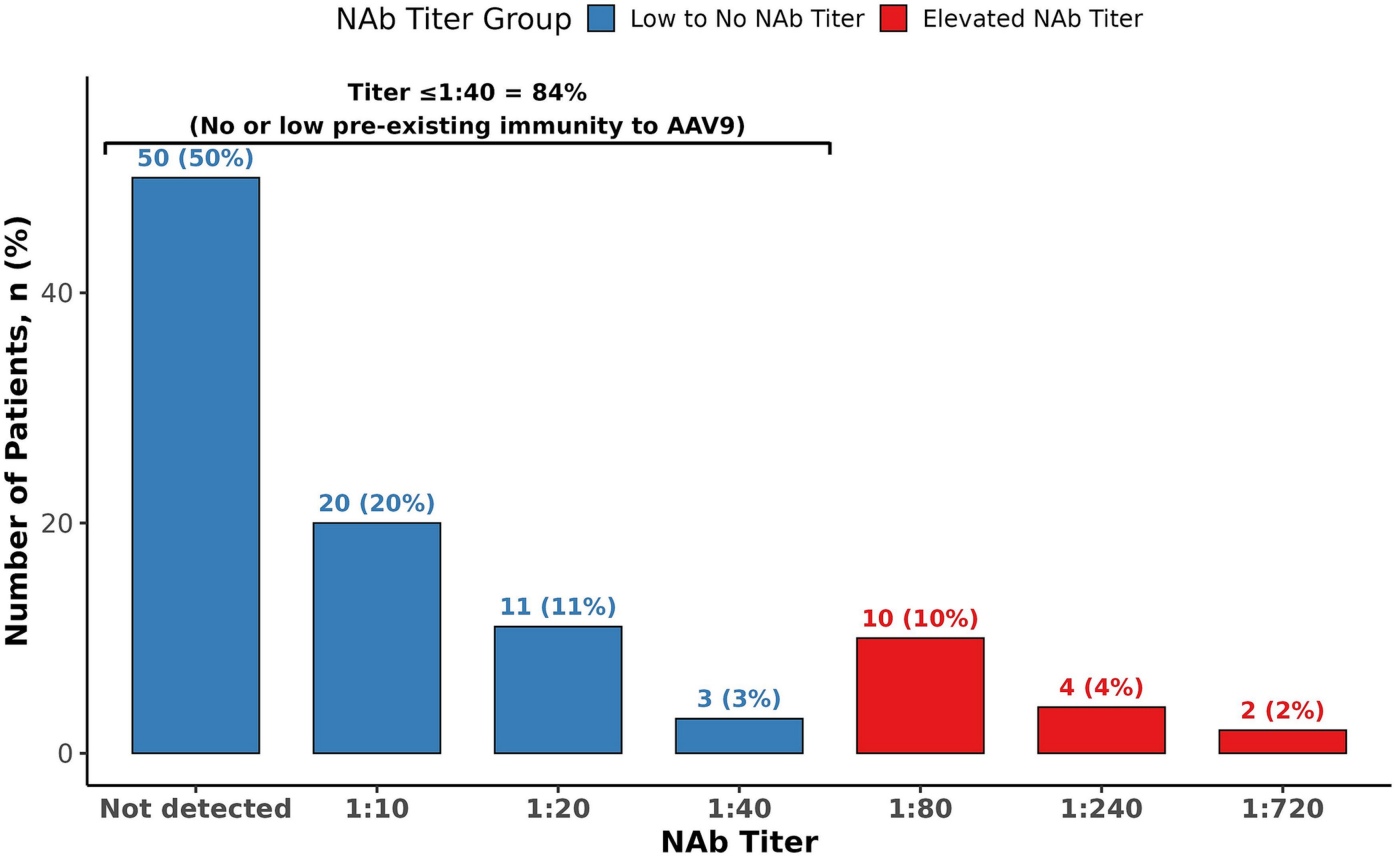
Distribution of neutralizing antibodies to AAV9.

**Figure 2:**
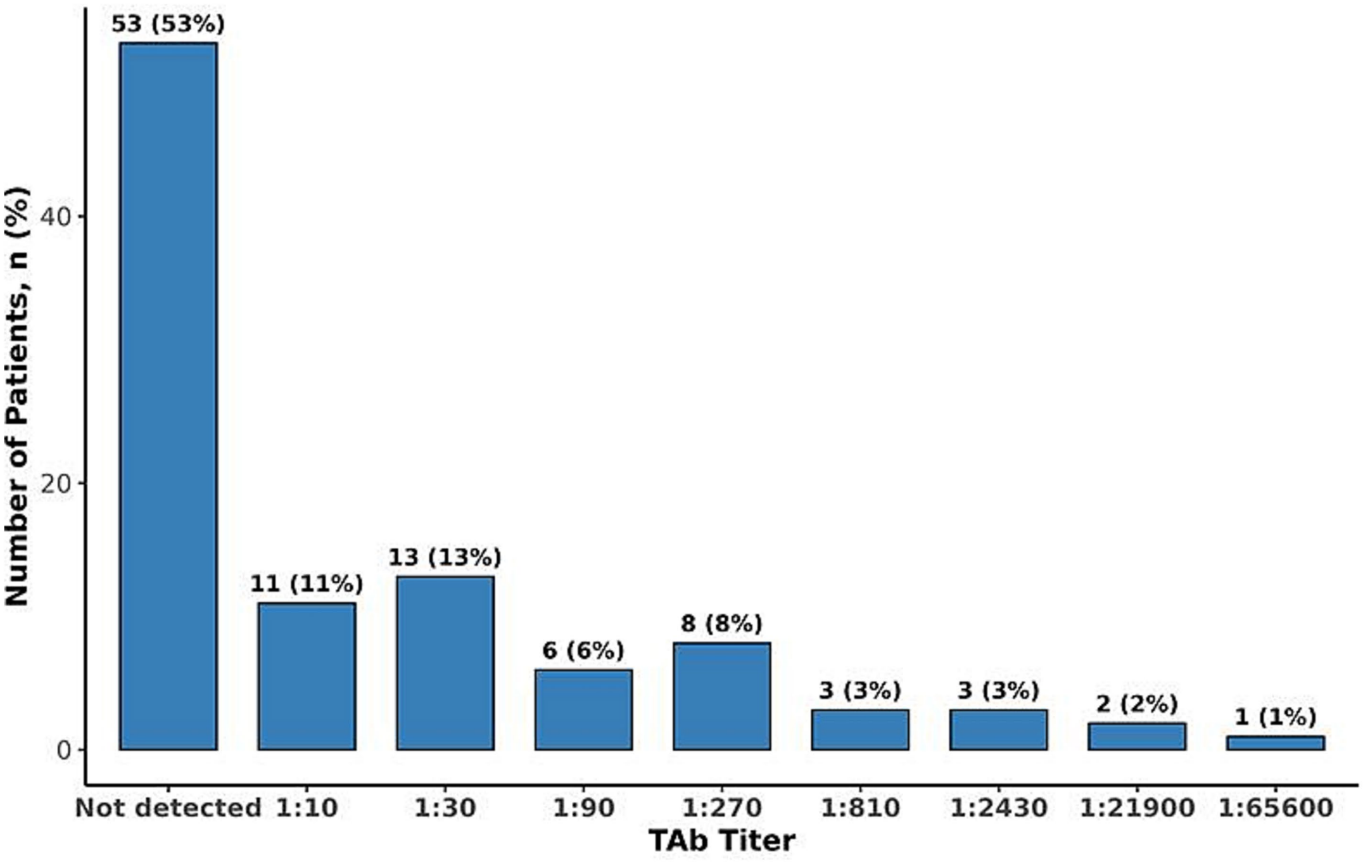
Distribution of total antibodies to AAV9.

NAb against AAV9 were detected in 50% of patients (N = 50). Among those with detectable NAb, titers ranged from 1:10 to 1:720 with a median of 1:20 (Figure 1). Notably, only 16% of patients exhibited titers exceeding 1:40, indicating a relatively low prevalence of high-titer responses. TAb against AAV9 were detected in 47% of patients (*N* = 47), with titers ranging from 1:10 to 1:65,600 and a median titer of 1:640 (Figure 2). A significant correlation was observed between TAb and NAb levels (*r* = 0.671, *p* < 0.001), indicating a high degree of concordance. This finding suggests that measuring AAV9 TAb titer could be a surrogate method to determine eligibility to receive an AAV9 gene therapy, as ECL-based assays for TAb are easier and more standardized to perform than transduction inhibition assays.

Exploratory subgroup analyses were conducted to evaluate potential associations between clinical characteristics and antibody levels. No meaningful differences in the prevalence of detectable NAb titers were observed by age (*p*-value = 0.791), sex (*p* = 0.398), or NYHA Class >II (*p* = 0.766). Similarly, prevalence of detectable TAb titers showed no differences by age (*p* = 0.917), sex (*p* = 0.666), or NYHA Class >II (*p* = 0.596) (all p = ns). Furthermore, no discernible trends existed across ethnic groups (*p* = 0.837 for NAb, *p* = 0.586 for TAb).

## Discussion

Findings from this seroprevalence study suggest that approximately half of the participants with *MYBPC3*-associated HCM exhibited detectable NAb or TAb titers to AAV9. Moreover, only 16% had NAb titers exceeding 1:40, a threshold relevant for TN-201 gene therapy eligibility, and only 6% had NAb titers exceeding 1:80, the maximum allowable AAV9 titer threshold in the TN-201 gene therapy trial. Gene therapy trials have prioritized enrolling patients with low anti-AAV NAb or TAb titers to maximize the likelihood of effective transgene delivery and efficacy while mitigating potential immune responses, despite minimal data linking adverse events to pre-existing antibodies, despite thousands treated with AAVs (13). Currently, there is a lack of standardization of assays that measure neutralizing activity and total antibodies; therefore, there is a variation in cut-off titer values where participants are considered positive. Previous studies set the cut-off for seropositivity at 1:50 (15, 16). It is also important to note that for previous studies, the cut-off was determined in healthy US donors (15, 17). In addition, the cut-off threshold for determining seroprevalence may change with geographical location and/or the utilization of patient-specific samples during validation of an assay. The MyPEAK-1 gene therapy clinical trial of TN-201 currently requires patients to have an AAV9 NAb titer ≤1:40 for inclusion into the study and may allow titers ≤1:80 in the future. Importantly, we found no association with patient age and serostatus, indicating that older subgroups are as likely to be seroeligible as younger cohorts. Sex, ethnicity, and NYHA class were not associated with NAb or TAb status, although prior studies have observed differences in serostatus by ethnicity (14, 18). This study population was predominantly White and exclusively adult and symptomatic, which may limit conclusions regarding differences in serostatus across all *MYBPC3*-associated HCM subgroups. Other relevant seroprevalence studies have shown comparable overall lower seropositivity rates for AAV9 compared to other serotypes, though several of these studies show that rates are higher among African American and Hispanic donors than other races/ethnicities (Table 2) (14–20). Importantly, several of these studies did not show increasing seropositivity over time.

**Table 2:** Baseline demographics and medical history of study population.

Study	Population	Sample size	NAb titer threshold	Ethnicity	AAV9 seropositive (%)
Verma et al. (18)	DMD	101	1:5	African American	59
				Southeast Asian Indian	50
				Hispanic	36
				Caucasian	32
Boutin et al. (15)	Healthy adults	226	1:20	Caucasian (France)	34
Mumiro et al. (17)	Healthy adults	85	1:14	Asian (Japan)	37
	Hemophilia	59		Asian (Japan)	27
Khatri et al. (14)	Healthy adults	100	1:5	African American	79
				Hispanic	50
				Caucasian	38
Navarro-Oliveros et al. (19)	Healthy adults	100	1:20	Caucasian (Spain)	17
Sierra-Delgado et al. (20)	Healthy adults	60	1:50	Hispanic (Colombia)	23
	Heart failure	60		Hispanic (Colombia)	20

In our study, the strong correlation between TAb and NAb titers suggests that TAb could become a reliable measure of pre-existing immunity. As more AAV gene therapy clinical trials are conducted, sponsors should advocate standardized screening assays and antibody titer cut-offs for future trials.

In conclusion, while approximately 50% of patients with *MYBPC3*-associated HCM had detectable anti-AAV9 antibody titers, 84% had low or no NAb titers to AAV9. The low prevalence of pre-existing immunity in this population indicates that most *MYBPC3*-associated HCM patients could be eligible for an AAV9 gene therapy, such as TN-201.

## Data Availability

The original contributions presented in the study are included in the article/supplementary material, further inquiries can be directed to the corresponding author/s.

## Generated AI statement

The authors declare that no Gen AI was used in the creation of this manuscript.

Any alternative text (alt text) provided alongside figures in this article has been generated by Frontiers with the support of artificial intelligence and reasonable efforts have been made to ensure accuracy, including review by the authors wherever possible. If you identify any issues, please contact us.
